# Tsetse distribution, trypanosome infection rates, and small-holder livestock producers’ capacity enhancement for sustainable tsetse and trypanosomiasis control in Busia, Kenya

**DOI:** 10.1186/s41182-020-00249-0

**Published:** 2020-07-31

**Authors:** Ferdinard Adungo, Tom Mokaya, Olipher Makwaga, Matilu Mwau

**Affiliations:** Kenya Medical Research Institute, P.O. Box 54840-00200, Nairobi, Kenya

**Keywords:** Tsetse density, Trypanosome infection rate, Trypanosomiasis, Vector control, Busia county

## Abstract

**Background:**

Tsetse flies are the cyclical vectors of both human and animal diseases. Kenya’s commitment to eradicate tsetse and trypanosomiasis dates to the 1980s through various control approaches which were spearheaded by the African Union. The aggressive control programmes together with climatic, land-use, and socio-economic changes immensely contributed to the reduction of African trypanosomiasis. Since 2012, Kenya has not recorded a case of human trypanosomiasis. However, African animal trypanosomiasis remains a major challenge to livestock production in 38 out of 47 counties. We aimed to determine the prevalence of tsetse flies and trypanosome infection rate and to build the capacity of small-holder livestock producers in vector control activities in Busia county.

**Methods:**

This cross-sectional study was conducted between May 2018 and December 2018 in Busia county, a beneficiary of the previous African Union-led trypanosomiasis and tsetse control initiatives. Odour-baited biconical traps were deployed for 48 h in five sampling areas. Captured tsetse flies were analysed by microscopy for trypanosome infections. Additionally, training and field demonstrations were conducted as part of capacity building to enhance participation of small-holder livestock producers in tsetse control activities.

**Results:**

A total of 94 tsetse flies mainly *Glossina fuscipes fuscipes* were captured from the five sampling areas. The apparent fly densities range from 0.08 to 1.55 tsetse per trap per day. Additionally, 75 biting flies mainly *Stomoxys* spp. were also trapped. An overall tsetse infection rate of 1.39% and 4.17% was observed for *Trypanosoma congolense* and *Trypanosoma vivax*, respectively. Regarding capacity building, a total of 26 small-holder livestock producers were trained on tsetse and trypanosomiasis control activities. Out of which, five were selected as focal persons and were further trained on integrated vector management techniques and tsetse survey methods.

**Conclusions:**

Our findings revealed the existence of trypanosome-infected tsetse flies which could potentially spread to other parts of the county. Training of small-holder livestock producers in tsetse and trypanosomiasis control activities should be supported and integrated in the county animal health and veterinary services. Given the observed low tsetse densities and trypanosome infection rates, the elimination of trypanosomiasis in Busia county is feasible.

## Background

Trypanosome parasites are mainly transmitted through bites of infected tsetse flies (*Glossina* spp.). To date, both human and animal trypanosomiasis affect the poor and marginalised populations of sub-Saharan Africa. Over the years, concerted efforts of the World Health Organization (WHO) and other stakeholders, such as national governments, non-governmental organisations, and pharmaceutical companies, have greatly contributed to the reduction of trypanosomiasis in Africa. This success has necessitated the current WHO target of eliminating human African trypanosomiasis (HAT) in endemic foci by the year 2030 [1–4].

In Kenya, tsetse flies are endemic in 38 out of 47 counties with negative impacts on human and livestock health, wildlife, and tourism [5, 6]. The Kenya Tsetse and Trypanosomiasis Eradication Council (KENTTEC), established in 2012, recognises distinct diverse zones or ‘tsetse fly belts’ in the country infested by eight tsetse fly species. Historically, cases of human trypanosomiasis were prevalent in the 1980s especially at the Lake Victoria basin tsetse belt [7]. Climate change and anthropogenic activities along with past trypanosomiasis and tsetse control initiatives are attributed to the suppressed *T. b. rhodesiense* infections in Siaya, Busia, and Bungoma counties [8–11]. Since 2009, Kenya has not reported autochthonous case of HAT apart from two cases detected in 2012 from European tourists returning from the Masai-Mara National Reserve [3, 12]. The Mara Reserve is located in the southwest of Kenya-Tanzania boundary and also constitutes the Serengeti Park. Although the risk of HAT in game parks and animal conservation areas is low, they remain important sources of tsetse exposure to travellers and tourists [12–15].

On the contrary, African animal trypanosomiasis (AAT) also called ‘nagana’ is still a major problem in Kenya. Several trypanosome species such as *T. b. brucei*, *T. congolense*, *T. vivax*, *T. simiae*, and *T. suis* affect a larger area of the country partly due to the extra risk of transmission by other biting flies. The disease continues to impose major constraints to livestock health and productivity [5, 16, 17].

### Past tsetse and trypanosomiasis control activities in western Kenya

In the1980s, the African Union-based Programme Against African Trypanosomiasis (PAAT) conducted one of the major trypanosomiasis and tsetse eradication campaigns in Kenya. Under the Farming in Tsetse-Controlled Areas (FITCA) regional programmes, five tsetse-infested districts in western Kenya—Bondo, Siaya, Busia, Teso, and Bungoma—benefited from the 1999–2004 control activities. Phase 1 of FITCA Kenya (first 4 years) aimed at improving livestock production and sustainable tsetse control. The activities focused on improving community awareness on the benefits of integrated crop and livestock production systems. Also, the project introduced appropriate technology approaches to sustainable tsetse control, i.e. using traps, targets, and cattle treated with synthetic pyrethroids.

Phase 2 focused on institutional strengthening and human resource capacity building. The district government extension staff and the community were to promote improved livestock practices and effective land-use systems to enhance agricultural output. The project activities involved education and training on the methods of tsetse control, operated by farmers themselves. It was envisaged that better land-use practices would decrease tsetse habitats. FITCA Kenya also emphasised private sector involvement in the delivery of extension messages to farmers.

Another trypanosomiasis and tsetse control programme in the Kenya was initiated in 2005 by the AU-based Pan-African Tsetse and Trypanosomiasis Eradication Campaign (PATTEC) [18]. The goal of the PATTEC initiative was to improve food security and alleviate poverty in tsetse-infested areas. The first phase of PATTEC which ended in 2012 targeted tsetse eradication in Meru/Mwea, Lake Victoria, and Lake Bogoria basin regions. The PATTEC activities in the Lake Victoria basin region were implemented in Busia, Teso, Bungoma, and Siaya districts. Similarly, the activities involved were as follows: tsetse fly control by use of traps, insecticide-treated targets, insecticide application on animals (pour on), and livestock protective fences. Additionally, monitoring of the vector populations and screening of animals were done to detect trypanosome infections in the intervention areas. Furthermore, farmers were supported to increase animal production by the introduction of exotic dairy animals and the establishment of livestock spraying groups.

### The Busia context

As mentioned earlier, Busia is in western Kenya within the Lake Victoria basin tsetse fly belt that includes other counties such as Bungoma to the north and Siaya to the southwest. It also borders Lake Victoria to the southeast and the Republic of Uganda to the west. About 890,000 people reside in Busia (1700 km^2^) [19], one of the smallest counties in Kenya. The county is dotted with forested riverbanks, patches of dense vegetation, and forested wetlands/swamps which are ideal habitats for tsetse flies [20, 21]. The agricultural sector contributes approximately 50% of the household income in Busia county with an estimated 254,000 livestock (cattle—150,000, goats—85,000, sheep—17,000, pigs—2000) in 2013 [22]. The practice of livestock grazing and watering along the rivers and open swampy areas is still common. However, some small-holder livestock producers practise zero grazing and tethering of livestock within their homestead. Furthermore, traders in this region prefer buying livestock for beef from the cattle markets of eastern Uganda, AAT endemic region. Given the large numbers of livestock involved, the potential risk of introduction of new trypanosomes and other animal diseases is high. Not long ago, cases of trypanocidal drug resistance to existing trypanocides were reported in Uganda [23–25]. Therefore, there is a need for regular monitoring and surveillance of African animal trypanosomiasis and resistance to trypanocide drugs in the region.

Over the past decade, Busia county has undergone rapid environmental changes, increased encroachment and use of wetlands and riverine for farming activities. Changes in land-use, deforestation, erosion, and loss of biodiversity may affect the prevalence, distribution of tsetse flies, and disease transmission dynamics. Current data on tsetse distribution and population density given the new epidemiological settings in the county is lacking. To substantially achieve reduction in AAT and to maintain a downward trend in disease incidence, training of small-holder livestock producers in vector control activities among other measures is paramount. This study are set out to determine the current tsetse distribution and trypanosome infection rate and to enhance local capacity in vector control activities.

## Methods

### Study design

This cross-sectional survey was conducted to determine the prevalence of tsetse flies in two sub-counties of Busia. Considering that all regions of the county (former Busia and Teso districts) received the previously interventions, a cross-sectional design with a purposive sampling approach was employed to assess the tsetse situation in the historical low- and high-burden disease areas. In addition, capacity building of the local community in tsetse control activities was done to enhance participation and for sustainability.

#### Site selection

Selection of sites was based on several factors such as proximity and connectedness to Uganda, past tsetse species distribution, and disease burden. Historically, trypanosomiasis in Busia and Teso districts was more prevalent at the Uganda border areas [17, 26, 27]. This was attributed to the possible transboundary transmission and tsetse re-invasion. In the border region, *G. pallidipes* was predominant in Teso north sub-county partly due to the connectedness to Uganda via forested hills. Also, Teso north had a lower AAT burden compared to the other six sub-counties. To determine the *G. pallidipes* density in Teso north, two sites, Kapesur (*G. pallidipes* predominant) and Ikapolok (low tsetse density), were included in the survey.

Next, Teso south which recorded a high AAT burden and mostly infested with *G. fuscipes fuscipes* was selected to determine the respective tsetse density. Similarly, Kwangamor (historically low burden) and Ngelechom (high burden) sites were sampled. The Obekai site was selected based on historical HAT cases in Teso south [7]. This selection strategy was applied to achieve the inclusion of both historical low- and high-burden sites and to cater for the fragmented distribution of tsetse species in the county.

### Study area

**Fig. 1:**
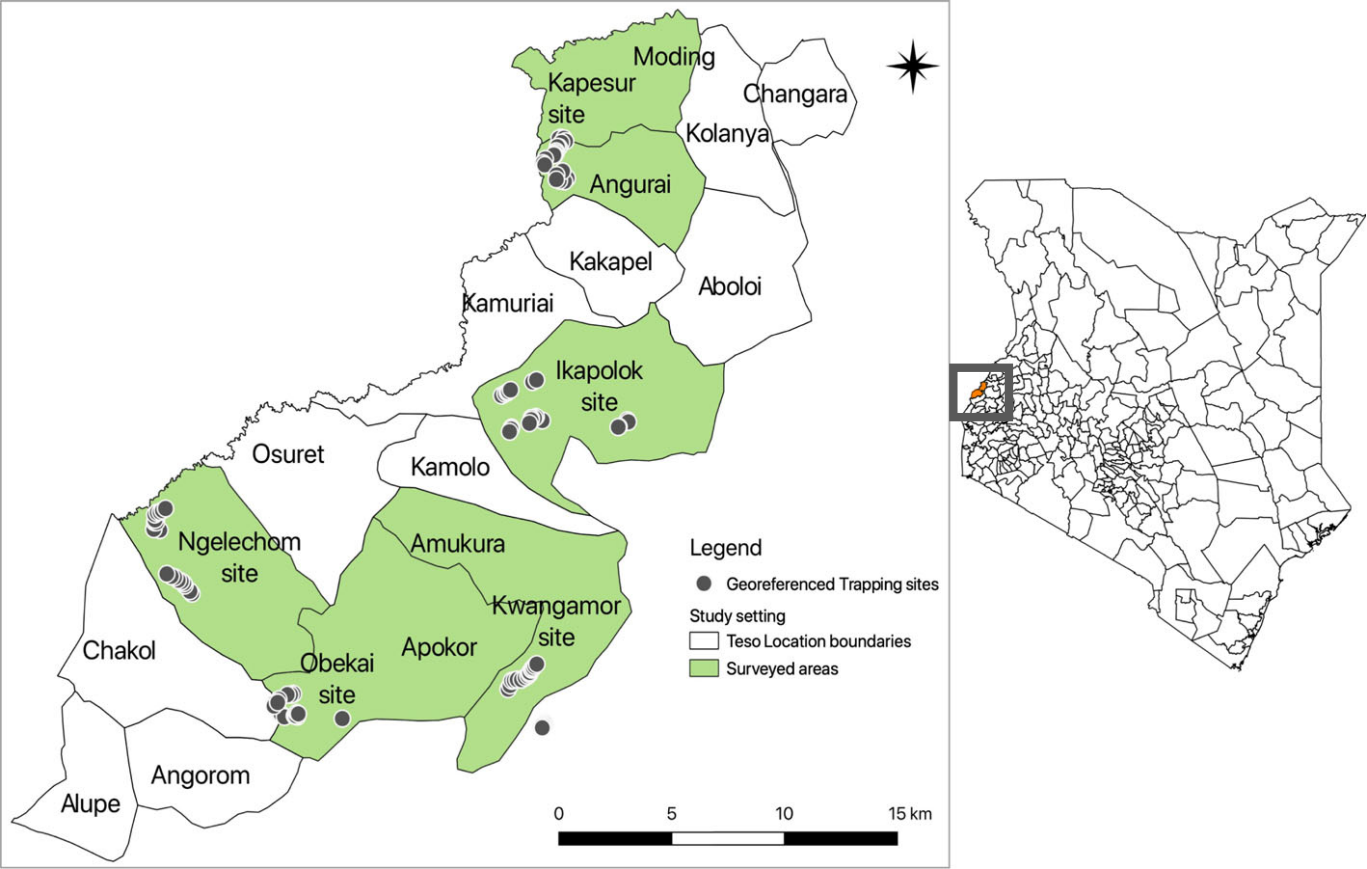
Map showing the study area and location of study sub-counties, and the selected sites for tsetse trapping. Dots represent the location of individual traps. The shaded area under the map shows the trapping sites

In 2010, Kenya abolished a centralised system of government and adopted a new constitution, which led to the creation of 47 counties. The current Busia county comprises two former administrative districts of Teso and Busia. This study was conducted in two sub-counties of Busia. Following the criterion described under the ‘Study design’ section, Teso south and Teso north were selected for the survey. The trapping sites were mapped out from five administrative villages, namely Kwangamor, Obekai, and Ngelechom in Teso south and Kapesur and Ikapolok in Teso north sub-county (Fig. 1).

### Community sensitisation and capacity building

Community sensitisation was done through the local assistant chief’s forum or ‘baraza’ where the research staff together with the local administrators conducted awareness sessions and distributed the flyers. Information and educational materials for the community were developed and translated into local language (Ateso). Permission to access private lands for tsetse trapping was sought from the people who attended the public gatherings. Subsequently, written informed consent for voluntary participation was obtained from the small-holder livestock producers who received training on tsetse control activities.

#### Selection of training participants

Training workshops were conducted in each village where selected small-holder livestock producers were sensitised as part of establishing local capacity in tsetse control activities. The selection of participants for the training was done by the area assistant chief. Each site independently selected participants who met the following criteria:

i. Having attained at least secondary school/O-level education.
ii. Be a small-holder livestock producer.
iii. Be interested in community-led trypanosomiasis and tsetse control activities.
iv. Available and willing to attend training.

Each site nominated five participants except Ngelechom, which had six because of the area size. Upon completion of the basic training, each site nominated a representative to form a team of five focal persons that received further training on basic tsetse biology and ecology, vector control techniques, baseline tsetse survey methods, trap deployment and servicing, interpretation of trap catches, and data collection. The training methods involved lectures, group discussions, and demonstrations using materials from previous entomological surveys. Furthermore, demonstrations and hands-on trainings in tsetse trapping and management of traps were done during the survey.

### Tsetse surveys

The tsetse survey was carried out from May 2018 to December 2018. All potential tsetse fly trap locations were mapped and geo-referenced by a global positioning system (GPS) prior to sampling (Fig. 1). The information on land-use, different ecological niches based on vegetation type, drainage, and human activities was recorded. A total of 20 odour-baited biconical traps placed 50–100 m apart were used for tsetse trapping in each site. The traps were baited by using phenol sachets, and bottles containing acetone were placed at the base. The traps were deployed for 48 h, checked, and emptied. The cages containing the flies were stored in cool boxes with moist cotton wool and shipped to the entomology laboratory for further analysis.

### Vector identification and dissection

In the laboratory, flies were immobilised in a cool box containing cotton wool soaked in ethyl acetate for about 5 min. The flies were counted, segregated, and subjected to morphological identification. Also, biting flies of entomological importance to trypanosomiasis transmission were recorded. To determine the presence of trypanosomes in the tsetse flies, all non-teneral flies were selected for dissection following standard procedures [28, 29]. Briefly, using a clean pair of forceps, the wings and legs of tsetse flies were removed. Under a dissecting microscope, the proboscis, salivary glands, and midguts were teased out. The dissected parts were placed on a slide with a drop of normal saline and observed under a compound microscope at × 100 and × 400 total magnification. Considering the differences in the lifecycles of trypanosome species, a presumptive identification of species was made based on the location of trypanosomes within the fly’s organs.

The following assumptions were applied:

i. *Trypanosoma vivax*: The trypanosomes form rosettes in the hypopharynx. The infective forms of *T. vivax* are only found in the mouthparts.
ii. *Trypanosoma congolense*: The trypanosomes develop in the midgut and infective forms migrate to the hypopharynx. The trypanosomes are found both in the intestines and in the mouthparts.
iii. *Trypanosoma brucei*: Have a complex lifecycle with procyclic forms found in the midgut, infective metacyclic forms in the salivary glands, and the hypopharynx. Trypanosomes are therefore found in the midgut, salivary glands, and mouth parts.

### Data analysis

Study data were recorded and stored in computer spreadsheets and analysed using simple descriptive summaries such as percentages and presented in tables and figures. The apparent tsetse density for each study area was determined based on the average number of tsetse flies caught per trap per day. Tsetse fly infection rate was determined as the proportion of tsetse flies positive for trypanosomes. All maps were created in *Quantum GIS 3.10 A Coruna* open source software.

## Results

### Local community participation and capacity building

A total of 26 small-holder livestock producers were trained on integrated vector management and trypanosomiasis control approaches. In addition, a team of five focal persons received further training on basic tsetse biology, vector control, and survey methods.

### Tsetse apparent density and trypanosome infection rates

Using odour-baited biconical traps deployed for 48 h, a total of 94 tsetse flies mainly *G. fuscipes fuscipes* were captured from the five sites. The apparent densities range from 0.08 to 1.55 tsetse per trap per day. Additionally, 75 biting flies mainly *Stomoxys* spp. were also trapped. The tsetse infection rate was 1.39% for *T. congolense* and 4.17% for *T. vivax* (Table 1).

**Table 1:** Summary of tsetse fly catches and *Trypanosoma* parasite positivity rates identified by dissection

Study site (village)	Traps with tsetse (%), *N* = 20	Total flies captured	Tsetse species	Identification of teneral and non-teneral				Apparent fly densities (FTD)^a^ (%)	Number dissected	Number with trypanosomes	Infection rate (%)	Trypanosome species
				Teneral male	Non-teneral male	Teneral female	Non-teneral female					
Kwangamor	19 (95)	62	*G. f. fuscipes*	9	13	3	37	1.55	50	1	1.39	*T. congolense*
										2	4.0	*T. vivax*
Obekai	4 (20)	6	*G. f. fuscipes*	0	2	0	4	0.15	6	1	0.17	*T. vivax*
Ngelechom	5 (25)	11	*G. f. fuscipes*	3	2	2	4	0.28	6	0	0.0	
Kapesur	3 (15)	3	*G. pallidipes*	0	1	0	2	0.08	3	0	0.0	
Ikapolok	6 (30)	12	*G. f. fuscipes*	2	2	0	8	0.30	10	0	0.0	

^a^Apparent tsetse density FTD = Σ*F*/(*T* × *D*) where FTD means the average fly count per trap per day, Σ*F* is the total number of captured flies, *T* is the total number of functional traps used, and *D* is the number of days (48 h) for which the traps were functional

**Fig. 2:**
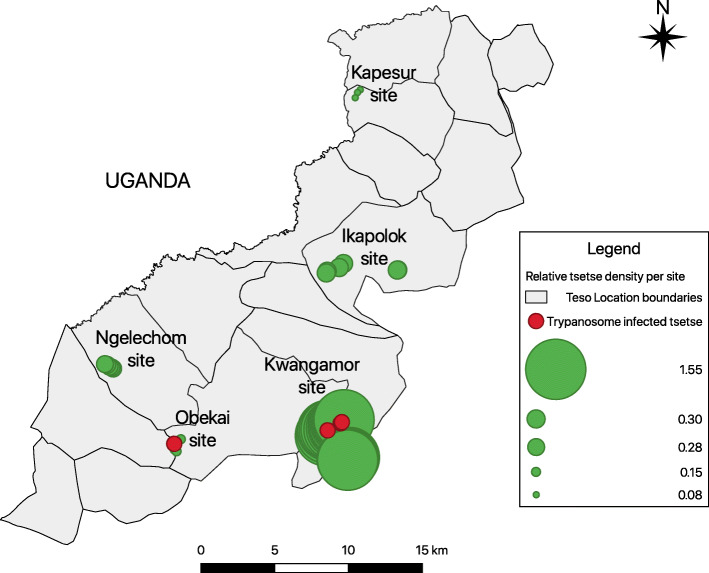
Map showing the apparent tsetse density and traps with infected tsetse fly. Colour codes: red shows at least one infected tsetse fly and green shows zero infected tsetse fly. Relative size of the circle shows differences in tsetse density at each site

In the three trapping sites of Teso south sub-county (Kwangamor, Obekai, and Ngelechom), only *G. fuscipes fuscipes* were caught. The Kwangamor site had the highest number of tsetse flies per trap per day. In Teso north sub-county (Kapesur and Ikapolok), both *G. pallidipes* and *G. fuscipes fuscipes* were captured. Kapesur was the only site where *G. pallidipes* were captured. All the other four sites were mainly infested with *G. fuscipes fuscipes* (Table 1). The *Stomoxys* spp. were the most predominant biting flies (92%) in the two sub-counties. Overall, the apparent tsetse densities range from 0.08 to 1.55 at Kapesur and Kwangamor sites, respectively. Regarding trypanosome infection rates, four of the seventy-five dissected tsetse flies had parasites. The location of traps that had at least one infected tsetse fly is shown in Fig. 2. The overall tsetse infection rate was 1.39% for *T. congolese* while that of *T. vivax* ranges from 0.17 to 4.0% for Obekai and Kwangamor sites, respectively.

## Discussion

Tsetse flies and trypanosomiasis mostly affect poor communities that are dependent on traditional methods of livestock keeping and small-scale crop production [25]. Most small-holder livestock producers in Busia county have for a long time relied on chemotherapy and chemoprophylaxis to maintain their livestock despite the constant threat from tsetse flies and other endemic diseases. Our findings showed that Busia county in general has a very low tsetse density and trypanosome infection rate. This could be attributed to the past control activities under FITCA and PATTEC, which promoted better land-use practices that could naturally decrease tsetse habitats. The activities are thought to have reduced tsetse densities by over 95% during the 1999–2012 intervention period [17, 30]. In addition, our findings showed *G. fuscipes fuscipes* as the most dominant tsetse species in the region accounting for over 97% of the catches. These results are consistent with the KENTTEC zoning of tsetse species in the country [9, 11, 31]. In landscapes that were ‘humanised’ or disrupted through cultivation, and clearance of bushes, tsetse flies were captured in traps set within crop fields. Previous research has reported similar phenomena [11, 26, 32, 33].

Majority of the tsetse flies captured in Teso south sub-county were *G. fuscipes fuscipes* with a small proportion (4.17%) found to be infected with *T. vivax*. Similarly, the infections with *T. congolense* were disproportionately lower (1.39%) in the sampled areas. Studies have shown *G. fuscipes fuscipes* to be a better vector for *T. vivax*, hence the higher infection rates among animals in this region [17, 27, 30, 31]. Furthermore, we observed a significant proportion of biting flies of the genera *Stomoxys* and *Tabanus* which could play a significant role in AAT epidemiology. Several studies have demonstrated mechanical transmission of *T. vivax* in cattle by the African tabanids [34, 35]. Because *T*. *vivax* is common in Busia county and could be transmitted by biting flies, vector control programmes should also target biting flies. Accordingly, the role of biting flies in the epidemiology of AAT in Busia and other parts of the country needs further investigation. To achieve the elimination of AAT in Busia, continuous monitoring and identification of residual tsetse breeding pockets and early deployment of control measures are crucial. Furthermore, the involvement of the small-holder livestock producers in control activities will help to renew commitment among stakeholders and to sustain the elimination effort.

## Conclusion

Our findings revealed the existence of trypanosome-infected tsetse flies which could potentially spread to other parts of the county. Training of small-holder livestock producers in tsetse and trypanosomiasis control activities should be supported and integrated in the county animal health and veterinary services. Given the low tsetse densities and trypanosome infection rates, the elimination of trypanosomiasis in Busia county is feasible.

## Study limitations

The trypanosome infections were determined by microscopy technique which is a less sensitive method. There is a possibility that some infected tsetse flies may have been missed leading to underreporting. Though not the goal of the study, parasitological analysis of animal samples would have enriched the findings.

Future studies will be designed to utilise molecular and serological techniques to analyse the parasites and vectors in detail.

## Data Availability

All data generated and analysed during this study are provided in this manuscript.

## Abbreviations

AAT: African animal trypanosomiasis
FITCA: Farming in Tsetse-Controlled Areas
FTD: Fly count per trap per day
GPS: Global positioning system
HAT: Human African trypanosomiasis
KENTTEC: Kenya Tsetse and Trypanosomiasis Eradication Council
PAAT: Programme Against African Trypanosomiasis
PATTEC: Pan-African Tsetse and Trypanosomiasis Eradication Campaign
